# Development of Alkaline Phosphatase-Fused Mouse Prion Protein and Its Application in Toxic Aβ Oligomer Detection

**DOI:** 10.3390/ijms232314588

**Published:** 2022-11-23

**Authors:** Kaori Tsukakoshi, Rikako Kubo, Kazunori Ikebukuro

**Affiliations:** Department of Biotechnology and Life Science, Graduate School of Engineering, Tokyo University of Agriculture and Technology, 2-24-16, Naka-cho, Koganei, Tokyo 184-8588, Japan

**Keywords:** Alzheimer’s disease, amyloid β, protein engineering, mouse prion protein, fusion protein, alkaline phosphatase

## Abstract

Amyloid β (Aβ) oligomers play a key role in the progression of Alzheimer’s disease (AD). Multiple forms of Aβ assemblies have been identified by in vitro and in vivo analyses; however, it is uncertain which oligomer is highly neurotoxic. Thus, understanding the pathogenesis of AD by detecting toxic Aβ oligomers is crucial. In this study, we report a fusion protein of cellular prion protein (PrPc) and alkaline phosphatase (ALP) from *Escherichia coli* as a sensing element for toxic Aβ oligomers. Since the N-terminus domain of PrPc (residue 23–111) derived from mice is known to bind to toxic Aβ oligomers in vitro, we genetically fused PrPc_23–111_ to ALP. The developed fusion protein, PrP–ALP, retained both the binding ability of PrPc and enzymatic activity of ALP. We showed that PrP–ALP strongly bound to high molecular weight (HMW) oligomers but showed little or no affinity toward monomers. The observation that PrP–ALP neutralized the toxic effect of Aβ oligomers indicated an interaction between PrP–ALP and toxic HMW oligomers. Based on ALP activity, we succeeded in detecting Aβ oligomers. PrP–ALP may serve as a powerful tool for detecting toxic Aβ oligomers that may be related to AD progression.

## 1. Introduction

Alzheimer’s disease (AD) is a neurodegenerative disorder characterized by the presence of senile plaques and neurofibrillary tangles in the brain of AD patient. The causative role of amyloid β (Aβ) peptide in AD is supported since the major constituent of senile plaques is the fibrillar form of Aβ [1]. Recent investigations have indicated that unlike monomers and fibrils, soluble Aβ oligomers are the primary toxic agents. Multiple forms of Aβ assemblies, from dimers to high molecular weight oligomers comprising more than 50 monomers, have been described in different studies, and some reports have demonstrated the neurotoxicity of specific oligomeric species [2,3,4,5,6]. However, it is uncertain which oligomer is the most neurotoxic and important for AD progression. Notably, a recent study by Hong et al. [7] suggested that the bulk of Aβ extractable from the AD brain is innocuous and only a small portion is highly toxic. These findings indicate the importance of analyzing a small pool of neurotoxic Aβ oligomers with specific ligands to understand AD pathogenesis.

Aβ oligomer-binding antibodies are widely used to detect Aβ oligomers. One possible mechanism by which these antibodies discriminate Aβ oligomers is the strong avidity of epitope-rich aggregates [8,9,10,11]. Thus, some antibodies bind not only to Aβ oligomers but also to less-toxic fibrils. Another mechanism involves conformational recognition. Some antibodies appear to recognize the conformation of Aβ oligomers, which are distinct from those of monomers and fibrils [12,13,14]. However, the relationship between the structures recognized by these antibodies and Aβ oligomer toxicity is poorly understood.

Here, we focused on cellular mouse prion protein (PrPc) as a ligand for toxic Aβ oligomers. Based on a genome-wide screening, mouse PrPc was identified as a natural neuronal receptor for Aβ oligomers that specifically bound to Aβ oligomers, but not to non-toxic oligomers, monomers, and fibrils [15,16]. The interaction between PrPc and Aβ oligomers has been reported to induce an array of AD features, including the activation of neurotoxic signaling pathways, loss of synapses, and inhibition of long-term potentiation [15,17,18]. Smith et al. [19] recently reported that PrPc shows the highest affinity for synthetic Aβ oligomers among dozens of reported Aβ oligomer receptors. Smith et al. showed that only PrPc strongly binds to soluble Aβ extracted from the human AD brain. Moreover, another study using brain extracts from various AD mouse models demonstrated that the amount of soluble Aβ detected with PrPc was tightly correlated with memory impairment in AD mice [20]. These observations highlight the importance of PrPc as a ligand for disease-relevant toxic Aβ oligomers. To date, for analyzing AD brain samples, PrPc has been applied as a capture ligand in sandwich ELISA by some research groups [17,19,20,21,22]. However, these assays require a pair of primary and secondary antibodies with sensitive reporter molecules for Aβ oligomer detection, which can complicate the entire assay.

In this paper, we provide the first report on enzyme-fused PrPc to achieve toxic Aβ oligomer detection in a simple and widely applicable manner. Construction of enzyme-fused PrPc by genetic technology allows for the preparation of a homogeneous molecule with a defined orientation and ligand/reporter ratio that can work as an ideal probe for obtaining reproducible results. To minimize the effect on enzymatic activity, we utilized the N-terminal domain of PrPc (residues 23–111) containing two Aβ oligomer binding sites. This PrPc domain has been shown to be soluble after expression in bacteria [23]. As a labeling enzyme, we utilized alkaline phosphatase (ALP). ALP is a homodimer protein; *Escherichia coli* (*E. coli*)-derived ALP is easily overexpressed in *E. coli,* and its mutant with D101S substitution shows increased activity [24]. In addition, because of their wide substrate specificity, ALP fusion ligands are applicable to a wide range of detection systems such as chromogenic detection, chemiluminescent detection, and electrochemical detection.

Our goal was to develop ALP-fused PrPc (PrP–ALP) that would be applicable to the toxic Aβ oligomer detection. We successfully demonstrated that PrP–ALP retained both the binding ability of PrPc and the enzymatic activity of ALP. PrP–ALP showed specificity for high-molecular-weight (HMW) Aβ oligomers. The observation that PrP–ALP neutralized the toxicity of Aβ preparations containing HMW oligomers suggests that it binds to HMW toxic Aβ oligomers. Additionally, we successfully detected Aβ oligomers using PrP–ALP enzymatic activity. Thus, PrP–ALP could be applied to the analysis of HMW toxic Aβ oligomers, which are considered to play an important role in AD progression.

## 2. Results

### 2.1. Preparation and Enzymatic Activity Analysis of PrP–ALP

**Figure 1:**
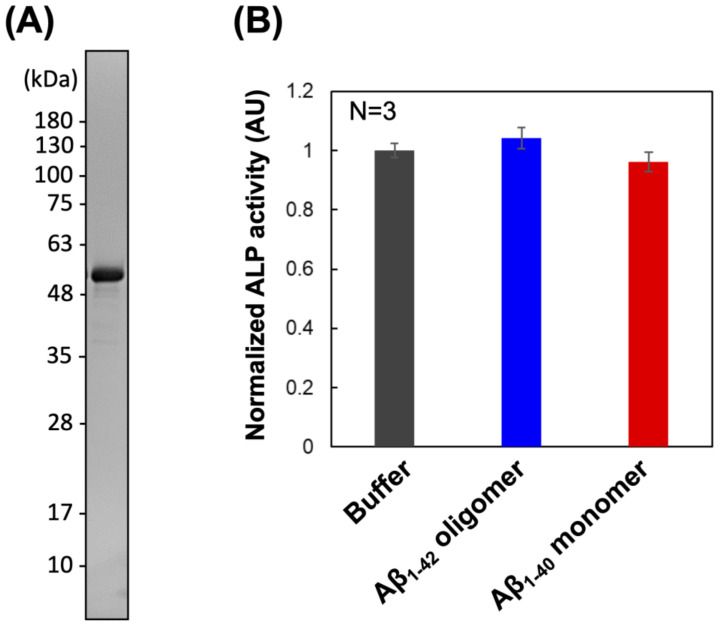
(**A**) SDS-PAGE analysis of PrP–ALP purified with affinity chromatography via StrepTag II. (**B**) Normalized enzymatic activity of PrP–ALP in the presence of Aβ oligomers. The data are shown as the mean ± SD in the graph (*n* = 3). Additionally, 100% of normalized ALP activity (AU) represents the ALP activity when incubated PrP–ALP with the solvent control (PBS buffer).

To construct PrP–ALP, the N-terminal sequence of mouse PrPc (residues 23–111) containing two Aβ oligomer binding sites (residues 23–27 amino acids and 95–110 amino acids) [15,23] was fused to the C-terminus of the D101S mutant of ALP from *Escherichia coli* (*E. coli*), which has been reported to show increased activity [24]. A StrepTag was added to the C-terminus of ALP via (GGGS)3 linker for protein purification. PrP–ALP was expressed in *E. coli* BL21 (DE3) cells and purified using affinity chromatography. SDS-PAGE analysis of the purified PrP–ALP revealed a single band at the predicted molecular weight (Figure 1A). The yield of the PrP–ALP was 150 U/L culture.

We compared the enzymatic activity of PrP–ALP with that of the D101S mutant ALP, which was also expressed in *E. coli* BL21 (DE3) cells. To investigate ALP activity, we calculated specific activity (U/nmol) from the coloration rate of *p*-nitrophenyl phosphate (*p*NPP). PrP–ALP showed 60% specific activity of the original ALP (Table 1). The N-terminal residues at the dimeric interface of ALP play a key role in the interaction of ALP monomers and stabilization of its quaternary structure [25], which may result in a slight reduction in PrP–ALP activity. We also evaluated the activity of PrP–ALP in the presence of Aβ. We used Aβ_1–42_ oligomers as the target Aβ aggregates for PrP–ALP (Figure 1B). PrP–ALP with a buffer or Aβ_1–40_ monomer was also prepared, and the enzymatic activity was compared with the addition of Aβ_1–42_ oligomers. Since Aβ_1–42_ easily aggregates in the buffer and Aβ_1–42_ monomers cannot be prepared, we used Aβ_1–40_, which is much less prone to aggregation than Aβ_1–42_, in the experiments to observe the effect of Aβ monomers on PrP–ALP. No significant change in ALP activity was observed in the presence of Aβ monomers or oligomers (Figure 1B). Overall, we considered that PrP–ALP retained sufficient enzyme activity for application in Aβ oligomer detection systems.

### 2.2. Evaluation of the Binding of PrP-ALP to Differently Aggregated Aβ

**Figure 2:**
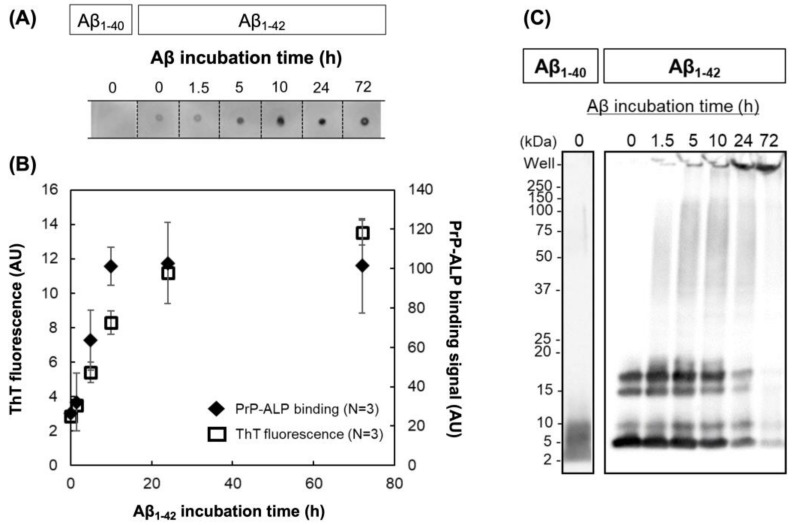
(**A**) Dot blotting of differently aggregated Aβ using PrP-ALP. The binding of PrP–ALP to Aβ oligomer was detected as the chemiluminescent signal generated from catalytic reaction of PrP–ALP. (**B**) Relationship between the PrP–ALP binding signals (closed diamond) and the ThT fluorescence (open square) from Aβ_1–42_. The data are shown as the mean ± SD in the graph (*n* = 3). (**C**) Western blot analysis of differently aggregated Aβ using anti-Aβ antibody, 82E1.

**Figure 3:**
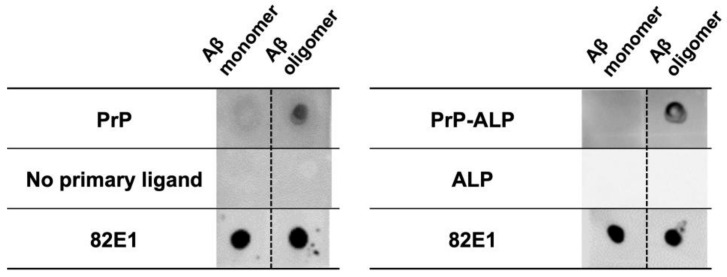
Dot blotting of Aβ using PrP, PrP–ALP, or ALP, respectively. The binding of PrP to Aβ was detected via StrepTag II fused to PrP, using HRP-conjugated anti-StrepTag II antibody. The PrP–ALP or ALP binding was detected based on their ALP activity.

During Aβ fibrillization, Aβ oligomers are generated by the aggregation of monomeric Aβ. Based on the dot blot assay, we analyzed the binding of PrP–ALP to the Aβ oligomers generated by in vitro incubation. Chemiluminescent signals generated by the PrP–ALP catalytic reaction were detected. When the Aβ samples were subjected to PrP–ALP, no spots were observed in the Aβ_1–40_ monomeric preparations. In contrast, for Aβ_1–42_ oligomeric preparations, spots were observed with increasing spot intensity in an Aβ_1–42_ incubation time-dependent manner. Spot intensities indicating PrP–ALP binding reached a plateau before the saturation of ThT fluorescence (Figure 2A, B). These results suggest that PrP–ALP bound to oligomeric Aβ species formed late during Aβ fibrillization. Western blotting of Aβ samples using an anti-Aβ N-terminus antibody, 82E1, demonstrated that Aβ_1–42_ first formed monomers or low molecular weight oligomers (LMW; 2~4-mer) and aggregated into high molecular weight oligomers (HMW; >20 kDa) after incubation (Figure 2C). The amount of Aβ_1–42_ monomers and LMW oligomers decreased, but that of HMW Aβ_1–42_ oligomers increased in a time-dependent manner. As the increasing levels of PrP–ALP binding signals correlated with the amount of HMW Aβ_1–42_ oligomers, PrP–ALP was considered to have binding selectivity toward HMW Aβ_1–42_ oligomers. Since ALP showed little or no affinity toward Aβ, PrP–ALP could bind to HMW Aβ_1–42_ oligomers via its PrPc sequence (Figure 3). PrPc has already been reported to show a high affinity toward HMW Aβ_1–42_ oligomers eluted in 158–670 kDa fractions of SEC [20]. Therefore, PrP–ALP seems to maintain the binding selectivity of the original PrPc. For the subsequent assay, we used Aβ_1–42_ oligomers prepared by incubation for 16 h, which contained HMW Aβ_1–42_ oligomers.

### 2.3. Aβ Oligomer Toxicity Neutralization with PrP-ALP

**Figure 4:**
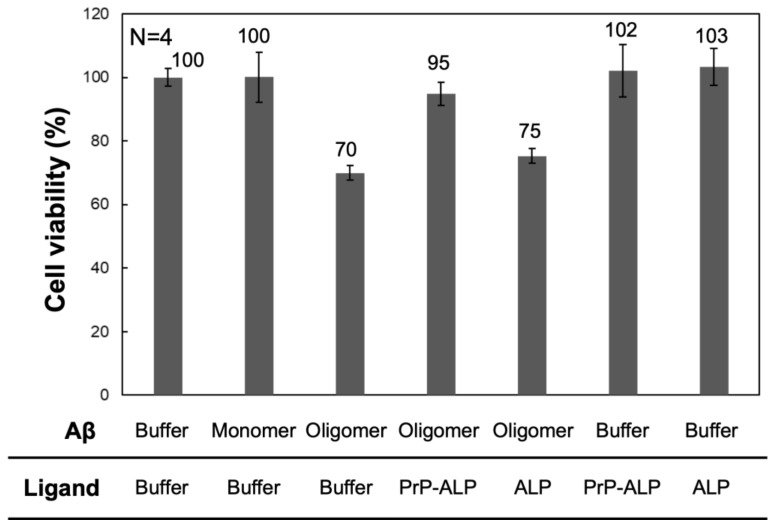
The cell viability measured with MTS assay after the addition of Aβ oligomers or Aβ monomers that had been pre-treated with PrP–ALP, ALP, or PBS buffer. We also used PBS buffer instead of addition of Aβ samples. The data are shown as the mean ± SD in the graph (*n* = 4).

To validate the interaction between PrP–ALP and a pool of toxic Aβ oligomers, we investigated whether PrP–ALP neutralizes the toxic effect of Aβ oligomers in SH-SY5Y neuroblastoma cells. The Aβ oligomer preparation used in this study was toxic to SH-SY5Y cells (Figure 4). In the presence of Aβ oligomers, a 30% decrease in cell viability was observed, whereas the monomeric preparation exhibited no effect on cell viability. The addition of ALP did not rescue the toxic effects of Aβ oligomers. However, upon addition of PrP–ALP, cell viability was significantly recovered, indicating that the inhibitory effect targeted toxic Aβ oligomers. Therefore, PrP–ALP seemed to bind to toxic Aβ oligomers and block the interaction with the cell membrane or cell surface receptor proteins to neutralize their cytotoxicity. Based on the observation that PrP–ALP strongly bound to HMW Aβ oligomers (Figure 2), some of the HMW Aβ oligomers to which PrP–ALP binds seemed to be highly toxic, and their toxicity was neutralized by interaction with PrP–ALP. Overall, we showed that PrP–ALP can be used for the detection of toxic HMW oligomers.

### 2.4. Aβ Oligomer Detection Based on Sandwich Plate Assay Using PrP–ALP

**Figure 5:**
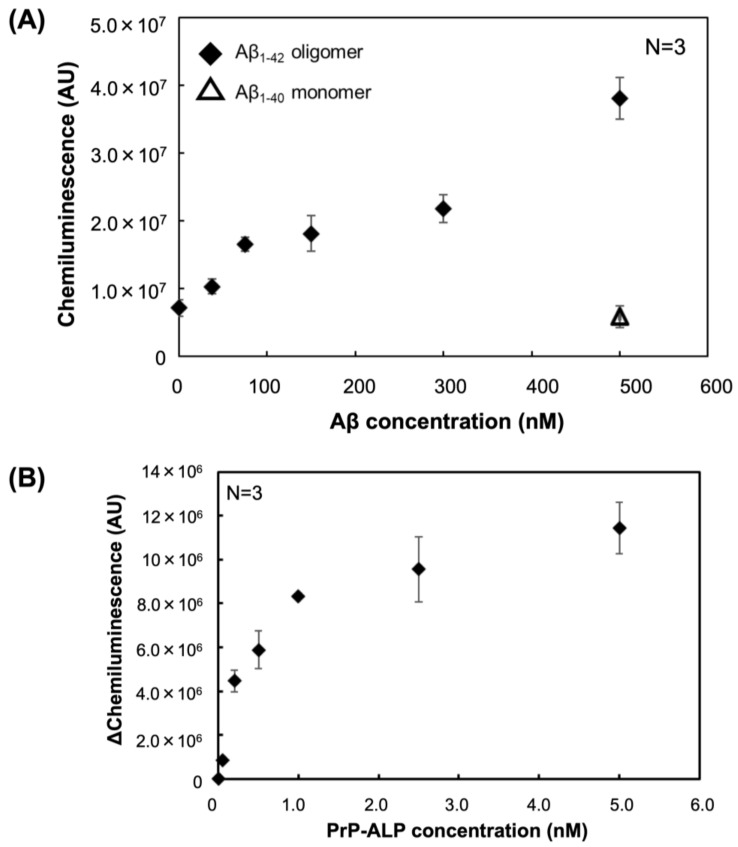
(**A**) Response chemiluminescent signals for Aβ_1–42_ oligomers (closed diamond) and Aβ_1–40_ monomer using a sandwich assay that consists of anti-Aβ antibody and PrP-ALP. (**B**) Evaluation of the dissociation constant of PrP-ALP toward Aβ oligomers with the sandwich assay. The data are shown as the mean ± SD in the graph (*n* = 3).

To verify whether the PrP–ALP constructed in this study can be applied for Aβ oligomer sensing, we performed a sandwich plate assay to detect Aβ oligomers. The anti-Aβ N-terminus antibody 82E1 was immobilized as a capture antibody, and PrP–ALP was used as the detection ligand. Detection using PrP–ALP showed increasing signals that were dependent on Aβ oligomer concentration (Figure 5A). In contrast, no increased signal was detected for Aβ monomers. Because PrP–ALP appeared to specifically bind toxic Aβ oligomers (Figure 4), the sandwich assay using PrP–ALP had high specificity toward toxic Aβ oligomers. As the exact molar concentration of the toxic HMW Aβ oligomer is unclear due to the difficulty in concentration measurement, we calculated the apparent limit of detection (LOD) for Aβ oligomers in this assay using the Aβ monomer-equivalent concentration. The apparent LOD for the Aβ oligomers in this assay was calculated to be 35 nM in Aβ monomer equivalents.

In addition, we determined the dissociation constant (*K*_D_) of PrP–ALP toward Aβ oligomers based on this sandwich assay. The *K*_D_ of PrP–ALP toward Aβ oligomers was calculated to be 560 pM at the monomer equivalent concentration (Figure 5B). Notably, the *K*_D_ value was considerably higher than the *K*_D_ value of PrPc_23–111_ for Aβ oligomers (17 nM) reported in another paper [23].

## 3. Discussion

Several methods for the detection of Aβ oligomers have been developed using mouse PrPc or the N-terminal peptide of PrPc, because PrPc can specifically bind to toxic Aβ oligomers [15,16,17,18]. However, all these detection systems require tag-fused PrPc and enzyme-labeled secondary antibody recognizing the tag for the sensitive detection of Aβ oligomers. If a direct fusion protein of an enzyme to PrPc could be developed, the sensitivity would be maintained, and the addition of a secondary ligand would be unnecessary in the assay. The reaction and washing conditions were also easier than those in the previous assay using the enzyme-labeled secondary antibody. Furthermore, multimerization of the Aβ oligomer binding site of PrPc by the dimer form of alkaline phosphatase (ALP) would make it possible to develop an Aβ oligomer ligand that has increased affinity from the avidity effect.

In this study, we developed ALP-fused prion protein (PrP–ALP) as a ligand for the detection of toxic Aβ oligomers. We focused on a 23–111 amino acid partial sequence of PrPc that contains the Aβ oligomer binding sites (23–27 amino acid and 95–111 amino acid) [15,16]. The molecular weight of the partial sequence is only 10 kDa; therefore, fusion of the partial sequence from PrPc would have a small negative effect on ALP activity. As expected, PrP–ALP retained both the Aβ oligomer-binding ability of PrPc and enzymatic activity of ALP (Figure 1, Table 1, Figure 2, and Figure 3). Additionally, our analysis of the binding property of PrP–ALP, combined with a western blotting assay for oligomeric Aβs, revealed that PrP–ALP might recognize HMW Aβ oligomers (Figure 2).

Previously, it was reported that the 23–111 amino acids of mouse PrPc suppress the toxicity of Aβ oligomers in mouse neurons [23]. Since toxic Aβ oligomers, to which PrP binds, have been reported to activate signaling involved in cytotoxicity via interactions with cell surface receptor proteins [15,17,18,26,27], it has been suggested that mouse PrPc inhibits toxicity-related Aβ oligomer-protein interactions by binding to Aβ oligomers. We performed a cell cytotoxicity neutralization assay to address the fact that PrP–ALP specifically recognizes toxic Aβ oligomers (Figure 4). As a result, pre-incubation of PrP–ALP with Aβ oligomers considerably neutralized the toxicity of Aβ oligomers but not ALP, as previously shown. Therefore, PrP–ALP also appeared to bind to HMW-toxic Aβ oligomers.

Finally, based on the ALP activity measurements derived from PrP–ALP, we succeeded in detecting Aβ oligomers (Figure 5A). PrP–ALP completely discriminated Aβ oligomers from monomers. Thus, using PrP–ALP, we achieved toxic Aβ oligomer detection in a simple way, without the use of additional ligands, which would complicate the steps of the whole assay. The sensitivity of the sandwich-type assay with an apparent LOD of 35 nM in Aβ monomer equivalents was demonstrated. Unfortunately, the exact molar concentration of toxic HMW oligomers is unknown, because it was challenging to purify the Aβ oligomers. Since the Aβ monomer-equivalent concentration was used in the LOD calculation, the number of LOD appeared large, but the western blotting results (Figure 2C) indicated that the amount of HMW oligomer should be small, so the actual LOD would be even lower. Since ALP can catalyze the dephosphorylation of various substrates and can be applied not only to chemiluminescence detection but also to highly sensitive electrochemical detection, we believe that LOD can be further improved by investigating suitable assay systems in the future.

Importantly, we also demonstrated a 30-fold improvement in the binding dissociation constant (*K*_D_) of PrP–ALP for Aβ oligomers (*K*_D_ = 560 pM, Figure 5B) compared to that of PrP alone as previously reported (*K*_D_ = 17 nM) [23]. Because ALP with enzymatic activity forms dimers, the fusion of PrP with ALP results in the formation of bivalent PrP on ALP. The binding ability of bivalent ligands (for instance, IgG) is stronger than that of monovalent ligands (e.g., fragment antibody). Furthermore, we have previously reported that the construction of bivalent aptamers and nucleic acid ligands for amyloidogenic protein oligomers can significantly improve the binding ability of aptamers [28]. Therefore, we considered that making PrP multivalent was the cause of the improved *K*_D_ value, and the fusion to ALP contributed not only to the simplification of the detection system, but also to the improvement of the ligand. Toxic Aβ oligomers might be dispersed in the brain of patients with AD or AD model mice, and the Aβ oligomers derived from the brain were expected to be present in very low concentrations. Therefore, PrP–ALP would be applicable to the measurement of brain-derived toxic Aβ oligomers.

## 4. Materials and Methods

### 4.1. Cloning, Expression, and Purification of PrP–ALP

The gene encoding 23–111 amino acids of mouse-derived PrPc was obtained by PCR amplification from an expression vector for mouse-derived full-length PrP [25]. The gene encoding *Escherichia coli*-derived ALP with a D101S substitution was fused to the 3′ end of the PrPc23–111 coding sequence by overlap extension PCR using a primer pair with StrepTag II. The PCR product was subcloned into the pET28a vector at *Nde*I and *Hin*dIII sites. Next, based on PCR for pET28a–PrP–ALP using two PCR primers to add the *Nde*I site at the 5′ end and a StrepTag II sequence with a linker sequence (GGGSGGGSGGGS) at the 3′ end, a PrP–ALP gene with StrepTag II was constructed. Finally, the gene was subcloned into the pET30c vector at *Nde*I and *Hin*dIII sites.

PrP–ALP was expressed in *Escherichia coli* BL21 (DE3) cells at 20 °C for 50 h with overnight express autoinduction (Novagen, WI, USA). The cells were harvested after 50 h and resuspended in cell lysis buffer (100 mM Tris-HCl, 500 mM NaCl, 1 mM MgCl_2_, 100 μM ZnCl_2_, and 4 mM 4-(2-aminoethyl) benzenesulfonyl fluoride hydrochloride, cOmplete™ ULTRA Tablet, pH8.0). The cell suspension was ultrasonicated four times for 30 s and centrifuged at 20,000 g for 30 min. The supernatant was collected and centrifuged at 113,300 g for 30 min. The supernatant was collected and filtered through a 0.45-mm nitrocellulose filter (ADVANTEC, Tokyo, Japan). PrP–ALP was affinity-purified using StrepTrap HP column (GE Healthcare, Little Chalfont, UK). The purity of protein samples was confirmed by sodium-dodecyl-sulfate–polyacrylamide gel electrophoresis (SDS-PAGE).

### 4.2. Enzymatic Activity Assay

The ALP activity of the purified PrP–ALP was measured using *p*-nitrophenyl phosphate (*p*NPP) as a substrate. The conversion of pNPP to *p*-nitrophenolate was monitored at 405 nm in a reaction buffer (1 M Tris-HCl, 150 mM NaCl, 1 mM MgCl_2_, 100 µM ZnCl_2_, pH 8.0) using a UV-1200 spectrometer (Shimazu Co., Kyoto, Japan). *V*_max_ and *K*_m_ values were calculated from Lineweaver–Burk plots.

### 4.3. Aβ Preparation

Synthetic Aβ_1–40_ and Aβ_1–42_ peptides (Peptide Institute, Inc., Osaka, Japan) were obtained as lyophilized powders. The peptides were reconstituted in 1,1,1,3,3,3-hexafluoro-2-propanol (HFIP) to a concentration of 0.5 mM. The peptide solution was evaporated to form a film and stored at −80 °C.

To prepare oligomeric Aβ, the Aβ_1–42_ peptide film was dissolved in dimethyl sulfoxide (DMSO) to a concentration of 5 mM. After sonication for 90 s, Aβ_1–42_ peptides were diluted to 100 μM in Ham’s F12 medium (IFP Co., Yamagata, Japan) and incubated at 22 °C. At each time point, aliquots were collected and mixed with 25 μM ThT (Sigma-Aldrich, St. Louis, MO, USA) solution. The ThT fluorescence intensities were monitored with excitation at 450 nm and emission at 486 nm. To prepare monomeric Aβ, the Aβ_1–40_ peptide film was dissolved in DMSO to a concentration of 1 mM and diluted in Ham’s F12 medium immediately before use.

### 4.4. Western Blotting Analysis for Prepared Aβ

Aβ preparations were separated by SDS-PAGE and transferred onto nitrocellulose membranes using a Trans-Blot Turbo transfer system (Bio-Rad, Hercules, CA, USA). The membrane was blocked with 4% (*w*/*v*) skim milk in TBS (50 mM Tris-HCl, 150 mM NaCl, 5 mM KCl, pH 7.4) containing 0.05% (*v*/*v*) Tween-20 (TBST) for 1 h at room temperature. After washing three times with TBST, the membrane was incubated with the anti-Aβ N-terminus antibody, 82E1 (epitope:1–16 aa of Aβ) solution for 1 h at room temperature. The membrane was washed three times with TBST and incubated with HRP-conjugated anti-mouse IgG (Promega, Madison, WI, USA) solution for 1 h at room temperature. Finally, chemiluminescent signals were detected using an LAS 4000 mini (GE Healthcare) with an Immobilon Western chemiluminescent HRP substrate (Merck Millipore, Billerica, MA, USA).

### 4.5. Dot Blot Assay to Evaluate PrP–ALP Binding Aβ Species

Aβ oligomers were spotted onto nitrocellulose membranes and dried for 20 min at room temperature. The membrane was blocked with 4% (*w*/*v*) skimmed milk in TBST for 1 h at room temperature. After washing three times with TBST, the membrane was incubated with 200 nM or 10 nM of PrP–ALP in TBST containing 1 mM MgCl_2_ and 100 μM ZnCl_2_ for 1 h at room temperature. After washing, the membranes were rinsed with TBST containing 1 mM MgCl_2_ and 100 μM ZnCl_2_ for 5 min at room temperature. Chemiluminescent signals were detected using LAS 4000 mini with CDP-*Star* ready-to-use substrate (Roche Diagnostics, Basel, Switzerland). To evaluate the binding of PrP-strep tag II, StrepMAB-Classic HRP conjugate (IBA Lifesciences, Goettingen, Germany) was used for detection.

### 4.6. Sandwich Plate Assay Using PrP–ALP to Detect Aβ Oligomers

A 96-well PolySorp flat-bottom plate (436111, Thermo Fisher Scientific K.K., Tokyo, Japan) was coated with 6 pmol of anti-Aβ antibody, 82E1 (epitope:1–16 aa of Aβ) in PBS buffer (137 mM NaCl, 2.7 mM KCl, 8.1 mM Na_2_HPO_4_, 1.4 mM KH_2_PO_4_, pH 7.4) and incubated overnight at 4 °C. After washing three times with TBST (0.05% Tween-20), the plates were blocked with 250 μL/well of 4% (*w*/*v*) skim milk in TBST for 1 h (r.t., 400 rpm). After washing three times with TBST, Aβ oligomers diluted in TBST were added to the microplates and incubated for 1 h (at 400 rpm). The plates were then washed three times with TBST and incubated with purified PrP-ALP in TBST containing 1 mM MgCl_2_ and 100 μM ZnCl_2_ for 1 h (r.t., 400 rpm). After washing seven times, chemiluminescence was measured using a BM chemiluminescent ELISA substrate AP (Roche Diagnostics). The *K*_D_ value of PrP-ALP for Aβ oligomers was calculated from Scatchard plot.

### 4.7. Cell Culture and MTS Cell Viability Assay

SH-SY5Y human neuroblastoma cells were routinely cultured in Dulbecco’s modified Eagle’s medium (D8062; Sigma-Aldrich) supplemented with 10% (*v*/*v*) fetal bovine serum at 37 °C in an atmosphere of 5% CO_2_ and 95% air. Cells were plated into a clear collagen-coated 96-well plate (0.5 × 10^3^ cells/well). Experiments were performed 24 h after plating. Cells were exposed for 48 h to 8 μM of Aβ oligomers that had been preincubated for 1.5 h with PrP–ALP, ALP, or PBS buffer. After removing the culture medium, cell viability was measured using the CellTiter 96^®^ AQueous One Solution Cell Proliferation Assay (MTS) system (Promega). The absorbance of the formazan product resulting from the reduction of MTS tetrazolium compound was recorded at 490 nm using a Multiskan FC plate reader (Thermo Fisher Scientific).

## 5. Conclusions

We developed PrP–ALP, the first ligand that directly fuses PrP with an enzyme for detection. PrP–ALP specifically binds to toxic Aβ oligomers with the improved *K*_D_ value of 560 pM at the Aβ monomer equivalent concentration. Because ALP was directly fused to PrP, the toxic Aβ oligomers would be detected without addition of other secondary ligands. Therefore, PrP–ALP would help to develop simple and useful biosensors. We believe PrP–ALP is a powerful tool for analyzing toxic HMW Aβ oligomers, which are considered to play an important role in AD progression.

## Figures and Tables

**Table 1:** Comparison of the kinetic parameter of enzymatic activity against *p*-nitrophenyl phosphate (*p*NPP) between the PrP–ALP and ALP (D101S).

	*K*_m_ (µM)	*V*_max_ (U/nmol)
PrP–ALP	75	3.9
ALP (D101S)	76	6.5

## Data Availability

Not applicable.

## References

[1] Hardy J., Selkoe D.J. (2002). The amyloid hypothesis of Alzheimer’s disease: Progress and problems on the road to therapeutics. Science.

[2] Lambert M.P., Barlow A.K., Chromy B.A., Edwards C., Freed R., Liosatos M., Morgan T.E., Rozovsky I., Trommer B., Viola K.L. (1998). Diffusible, nonfibrillar ligands derived from Abeta1–42 are potent central nervous system neurotoxins. Proc. Natl. Acad. Sci. USA.

[3] Walsh D.M., Tseng B.P., Rydel R.E., Podlisny M.B., Selkoe D.J. (2000). The oligomerization of amyloid beta-protein begins intracellularly in cells derived from human brain. Biochemistry.

[4] Gong Y., Chang L., Viola K.L., Lacor P.N., Lambert M.P., Finch C.E., Krafft G.A., Klein W.L. (2003). Alzheimer’s disease-affected brain: Presence of oligomeric A beta ligands (ADDLs) suggests a molecular basis for reversible memory loss. Proc. Natl. Acad. Sci. USA.

[5] Shankar G.M., Li S., Mehta T.H., Garcia-Munoz A., Shepardson N.E., Smith I., Brett F.M., Farrell M.A., Rowan M.J., Lemere C.A. (2008). Amyloid-beta protein dimers isolated directly from Alzheimer’s brains impair synaptic plasticity and memory. Nat. Med..

[6] Noguchi A., Matsumura S., Dezawa M., Tada M., Yanazawa M., Ito A., Akioka M., Kikuchi S., Sato M., Ideno S. (2009). Isolation and characterization of patient-derived, toxic, high mass amyloid beta-protein (Abeta) assembly from Alzheimer disease brains. J. Biol. Chem..

[7] Hong W., Wang Z., Liu W., O’Malley T.T., Jin M., Willem M., Haass C., Frosch M.P., Walsh D.M. (2018). Diffusible, highly bioactive oligomers represent a critical minority of soluble Abeta in Alzheimer’s disease brain. Acta Neuropathol..

[8] Yang T., O’Malley T.T., Kanmert D., Jerecic J., Zieske L.R., Zetterberg H., Hyman B.T., Walsh D.M., Selkoe D.J. (2015). A highly sensitive novel immunoassay specifically detects low levels of soluble Abeta oligomers in human cerebrospinal fluid. Alzheimers Res. Ther..

[9] Sevigny J., Chiao P., Bussiere T., Weinreb P.H., Williams L., Maier M., Dunstan R., Salloway S., Chen T., Ling Y. (2016). The antibody aducanumab reduces Abeta plaques in Alzheimer’s disease. Nature.

[10] Arndt J.W., Qian F., Smith B.A., Quan C., Kilambi K.P., Bush M.W., Walz T., Pepinsky R.B., Bussiere T., Hamann S. (2018). Structural and kinetic basis for the selectivity of aducanumab for aggregated forms of amyloid-beta. Sci. Rep..

[11] Jin M., O’Nuallain B., Hong W., Boyd J., Lagomarsino V.N., O’Malley T.T., Liu W., Vanderburg C.R., Frosch M.P., Young-Pearse T. (2018). An in vitro paradigm to assess potential anti-Abeta antibodies for Alzheimer’s disease. Nat. Commun..

[12] Kayed R., Head E., Thompson J.L., McIntire T.M., Milton S.C., Cotman C.W., Glabe C.G. (2003). Common structure of soluble amyloid oligomers implies common mechanism of pathogenesis. Science.

[13] Lambert M.P., Velasco P.T., Chang L., Viola K.L., Fernandez S., Lacor P.N., Khuon D., Gong Y., Bigio E.H., Shaw P. (2007). Monoclonal antibodies that target pathological assemblies of Abeta. J. Neurochem..

[14] Sebollela A., Cline E.N., Popova I., Luo K., Sun X., Ahn J., Barcelos M.A., Bezerra V.N., Lyra E.S.N.M., Patel J. (2017). A human scFv antibody that targets and neutralizes high molecular weight pathogenic amyloid-beta oligomers. J. Neurochem..

[15] Lauren J., Gimbel D.A., Nygaard H.B., Gilbert J.W., Strittmatter S.M. (2009). Cellular prion protein mediates impairment of synaptic plasticity by amyloid-beta oligomers. Nature.

[16] Chen S., Yadav S.P., Surewicz W.K. (2010). Interaction between human prion protein and amyloid-beta (Abeta) oligomers: Role OF N-terminal residues. J. Biol. Chem..

[17] Um J.W., Nygaard H.B., Heiss J.K., Kostylev M.A., Stagi M., Vortmeyer A., Wisniewski T., Gunther E.C., Strittmatter S.M. (2012). Alzheimer amyloid-beta oligomer bound to postsynaptic prion protein activates Fyn to impair neurons. Nat. Neurosci..

[18] Um J.W., Kaufman A.C., Kostylev M., Heiss J.K., Stagi M., Takahashi H., Kerrisk M.E., Vortmeyer A., Wisniewski T., Koleske A.J. (2013). Metabotropic glutamate receptor 5 is a coreceptor for Alzheimer abeta oligomer bound to cellular prion protein. Neuron.

[19] Smith L.M., Kostylev M.A., Lee S., Strittmatter S.M. (2019). Systematic and standardized comparison of reported amyloid-beta receptors for sufficiency, affinity, and Alzheimer’s disease relevance. J. Biol. Chem..

[20] Kostylev M.A., Kaufman A.C., Nygaard H.B., Patel P., Haas L.T., Gunther E.C., Vortmeyer A., Strittmatter S.M. (2015). Prion-Protein-interacting Amyloid-beta Oligomers of High Molecular Weight Are Tightly Correlated with Memory Impairment in Multiple Alzheimer Mouse Models. J. Biol. Chem..

[21] Freir D.B., Nicoll A.J., Klyubin I., Panico S., Mc Donald J.M., Risse E., Asante E.A., Farrow M.A., Sessions R.B., Saibil H.R. (2011). Interaction between prion protein and toxic amyloid beta assemblies can be therapeutically targeted at multiple sites. Nat. Commun..

[22] Nicoll A.J., Panico S., Freir D.B., Wright D., Terry C., Risse E., Herron C.E., O’Malley T., Wadsworth J.D., Farrow M.A. (2013). Amyloid-beta nanotubes are associated with prion protein-dependent synaptotoxicity. Nat. Commun..

[23] Fluharty B.R., Biasini E., Stravalaci M., Sclip A., Diomede L., Balducci C., La Vitola P., Messa M., Colombo L., Forloni G. (2013). An N-terminal fragment of the prion protein binds to amyloid-beta oligomers and inhibits their neurotoxicity in vivo. J. Biol. Chem..

[24] Zhang X.-E., Zhou Y.-H., Zhang Z.-P., Xu H.-F., Shao W.-H., Cass A.E.G. (2002). Engineering, *E. coli* Alkaline Phosphatase Yields Changes of Catalytic Activity, Thermal Stability and Phosphate Inhibition. Biocatal. Biotransform..

[25] Ogasawara D., Hasegawa H., Kaneko K., Sode K., Ikebukuro K. (2007). Screening of DNA aptamer against mouse prion protein by competitive selection. Prion.

[26] Smith L.M., Strittmatter S.M. (2017). Binding Sites for Amyloid-beta Oligomers and Synaptic Toxicity. Cold Spring Harb. Perspect. Med..

[27] Ohnishi T., Yanazawa M., Sasahara T., Kitamura Y., Hiroaki H., Fukazawa Y., Kii I., Nishiyama T., Kakita A., Takeda H. (2015). Na, K-ATPase alpha3 is a death target of Alzheimer patient amyloid-beta assembly. Proc. Natl. Acad. Sci. USA.

[28] Tsukakoshi K., Ikuta Y., Abe K., Yoshida W., Iida K., Ma Y., Nagasawa K., Sode K., Ikebukuro K. (2016). Structural regulation by a G-quadruplex ligand increases binding abilities of G-quadruplex-forming aptamers. Chem. Commun..

